# The Family of Araneides, or Spiders—Their Natural History—the Effects and Treatment of Their Bite—and the Use of Their Web as an Article of the Materia Medica

**Published:** 1848-05

**Authors:** Charles A. Hentz

**Affiliations:** Alabama


### WESTERN JOURNAL

OF

### MEDICINE AND SURGERY.

MAY, 1848.

ART. I.—*The Family of Araneides, or Spiders—their Natural History—the Effects and Treatment of their Bite—and the Use of their Web as an Article of the Materia Medica.* By CHARLES A. HENTZ, M.D., of Alabama. (Being an Inaugural Dissertation, submitted to the Trustees and Medical Faculty of the University of Louisville, February 1848, for the Degree of M.D.)

The Book of Nature is the great text-book of the medical man; spread out before him in rich profusion, it presents an unbounded field for unceasing observation and ennobling study.

First of all stands man—"the noblest work of God",—from the admirable and complex organization of whose physical structure, we descend by harmonious gradations, to the mite too small to be seen by the unassisted eye. At none of these steps in the scale of creation is it unprofitable to pause and observe, reaping as the rich reward of our observation and research, an exhibition of the same creative skill, the same adapting Providence, which directs the varied functions of the human system.

In the study of the humble Spider we will not be unrewarded, for besides having displayed to us the beautiful adaptation of structure to the performance of function, and of instinct to the mode of life intended by nature, there are practical bearings connected with the subject which are not unworthy our attention.

Commencing our study anatomically then:—The body of the spider is divided into the thorax and abdomen; the head being consolidated with the former, the united segments bear the name of *cephalothorax.*

The legs are eight in number, composed of five joints, and united to the cephalothorax by a short double joint, called the *coxa.* There is a curious surgical and physiological fact relating to the legs, which we must allude to in this place.

If the limb be cut off with a sharp instrument, luxated or fractured, the spider soon dies from a hemorrhage or escape of the vital fluid which nature cannot staunch; if, however, the leg be pulled or torn off from its articultion with the coxa, which is always the case, when the spider is held by one of its extremities, and left at liberty to struggle for escape, no pain or inconvenience seems to be experienced—but little fluid is lost, and the spider soon returns to its pursuits with its wonted agility. The leg moreover, when torn off at this joint, is soon reproduced, re-appearing at the moulting period, at first slender and small, progressively increasing till the whole attains its natural size and strength.

The eyes are *six* or *eight* in number, situated upon the anterior, superior part of the cephalothorax, and varying in different genera, in their mode of arrangement, constituting thus a distinguishing characteristic in the classification of these genera. Microscopically examined, each eye is found to be composed of a *cornea;* a *crystalline lens;* a *vitreous body;* a *kind of chamber* containing an aqueous humor; a *choroid coat;* and a *retina.*

With this short view of some of the external characteristics of the spider, let us consider some of its internal organs or systems by which the functions are carried on. And first of the

*Digestive System.*—The food of the araneides consists, for the most part, of the insects which they entrap in their webs or otherwise, and from which they abstract the vital fluid, whilst yet in a living state.

The mouth constituted for this kind of prehension, consists first, of two mandibulæ, opposed to each other, and possessed of a motion of apposition; these support, each, a hooked claw or fang, which is joined by a very free ginglymoid articulation, and has near its extremity a small perforation for emitting a poisonous fluid, which is secreted by a small gland lodged in an interspace of the muscles of the thorax, which gland is connected with this perforation by a fine excretory duct.

There are two maxillæ consisting, each, of a flattened lobe, and a lateral appendage, which supports the *palp* or *feeler*. At the junction and base of these maxillæ, is a *labium* articulated with the sternum.

When the prey is caught and secured, these fangs, after having given vent to their poison, to quiet the struggles of the victim, puncture and lacerate it, so as to let out the nutritious fluids which are squeezed out by the lateral or opposing motion of the maxillæ and conveyed through three pharyngeal orifices, into a broad and short œsophagus,—this empties into a stomach, or as M. Audouin terms it, "a gizzard," which is composed of four sacs, from which the alimentary canal is continued in the form of a straight, slender tube. We have here a confirmation of the general rule, with regard to the relative length of the intestines in herbivorous, and in carnivorous animals, for in the spider which is eminently of the carnivorous order, it is always short, and never convoluted, as is the case in many herbivorous insects.

This small intestine terminates in a dilated portion, to which is adherent externally, a kind of epiploon, filled with adipose granules; the dilated part gradually contracts, and then undergoes a second dilatation before its termination. Opening into the lower portion is a pouch in which terminate vessels which are termed biliary vessels, though this is a strange location for the biliary apparatus, being in all superior orders of animals, even in other insects, attached to the anterior or chylopoietic portion of the alimentary canal.

The fatty granules which are attached to the dilated portion of the intestine constitute the chief portion of the abdominal contents, and exhibit a beautiful provision of nature, for the exigencies of the spider, in affording a fund of nourishment during the long season of hibernation, or during the long seasons that sometimes occur, when they do not entrap any food.

The study of the *circulatory and respiratory systems*, will be found interesting.

The heart consists of a long vessel situated in the median line, upon the back of the abdomen, and held in its situation by small ligaments or muscles. It distributes vessels to all portions of the body, and in addition to these arteries, has two vessels of a larger size than the rest, attached by each extremity to the central vessel or heart and communicating by means of numerous minute ramifications with the pulmonary branchæ or respiratory apertures which exist beneath the body. There are no veins, they being replaced by irregular cavities or sinuses, existing between the different organs of the body. The blood being sent out by the heart, is distributed as in the higher orders of animals, by means of the arteries, to the different organs of the body; becoming venous, it is collected into the sinuses above described, whence finding its way to the pulmonary or branchial apertures, it becomes arterialized and returns back to the heart by the vessels above described, which are dignified by the name of *branchio-cardiac* vessels.

There is an apparatus peculiar to the spider, and very beautiful in its structure and operation—viz: that for secreting and emitting the fluid which, concreting in the air, forms the web.

The organ in which it is secreted exists in the abdomen and is formed of tortuous vessels, united irregularly, and varying in number and size—consisting essentially of two larger trunks and two smaller ones, recruited near their termination by a large number of small canals. The fluid secreted here passes out through spinnarets situated at the extremity of the abdomen, two long ones which are generally considered not to emit the fluid, but examined under a powerful microscope, distinct filaments of thread have been seen issuing from their surface; they are articulated and filiform—the others are four in number, short, and exhibiting on their surface a peculiar structure. The surface of each is perforated by an innumerable number of exceedingly minute apertures, through which the fluid passes as through a seive, hardening immediately on coming into the atmosphere, thus making as it is drawn out, the silken thread; these little filaments are of imperceptible fineness, till united together they form the cord, which from its extreme tenuity, we would scarcely conceive to consist of hundreds, nay, thousands of these primitive threads.

In securing its struggling victim, the spider, at the outset of the attack, or when the exertions of the insect are about to free it from the entangling threads, throws out a large cord or mass of this fluid uncoagulated, which wrapping around the limbs of the doomed victim, like the bird-lime on the plumes of the unfortunate sparrow, renders futile its most desperate writhings, and makes it an easy prey.

Having thus taken a short view of the principal anatomical characteristics of the spider, we will dwell for a while upon those characteristics which are less general—which serve to distinguish the different genera from each other. M. Walckenaer has made the following sub-division of the family—the classification being governed by the various habitudes and modes of life of the different genera, which genera are distinguished from each other by the arrangement of the eyes, form of the mouth, &c.

### ARANEIDES.

Mandibulæ moving horizontally—

*Mygale*, *Oletera*, and *Filistata*—living in holes and fissures—8 eyes.

Mandibulæ articulated vertically, moving laterally—

*Dysdera* and *Pylarus*—inclosing themselves in silken tubes—6 eyes, anterior,

*Lycosa*, *Dolemedes*, *Ctenus*, and *Oxyopes*, running swiftly to catch their prey; *Attus*, *Lyssomanes*, and *Synemosyna*—leaping and springing with agility to catch their prey—8 eyes, anterior and lateral, very unequal in size.

*Thomisus* and *Clubiara*, running and walking sideways and backwards; going abroad making web for nests; *Pholcus* (6 eyes) and *Spermophora*, going abroad, spreading long threads, making loose webs; *Tegenaria* and *Alagena*, spinning great webs of close texture like hammocks; *Herpyllus* wandering; *Epeira*, *Phyllira*, and *Tetraguatha*, spinning orbicular webs; *Mimetus*, wandering, predatory, taking the web of its victim; *Linyphia*, spreading regular webs of open mesh-work; *Theridion*, spreading irregular webs of open mesh-work—8 eyes, anterior, almost equal in size.

This list contains all the genera most commonly met with in the southern part of our country, to which my limited sphere of observation has been confined. Several of them are not found in Walcknaer's table, having been adopted by my father, who has been for many years engaged in this department of natural history—the result of whose observations is now being published in the Boston Journal of Natural History.

The characteristic instincts, habitudes and artifices of each genus, form an exceedingly interesting subject for observation—exhibiting the same beautiful adaptation of means to an end, the same fitness of organization to the mode of life in view that characterizes all of Nature's works.

The *Mygale* and *Oletera*, which are of stout limb and sturdy organization, make their houses in the bosom of their mother earth, burrowing with mathematical precision, deep, round holes, which they line with an exquisitely delicate texture of finely woven silk, and cover the whole with a door or lid of trash, dirt, &c., hinged on with a silken lash, and fitting to the raised margin of the hole with the most perfect aptitude.

When alarmed or attacked, the spider, fixing a firm hold on the silken lining of his tube, seizes the lid with his anterior feet and with determined grasp, bids defiance to all intruders.

The *Dysdera* and *Pylarus* are as ingenious artizans as the preceding—selecting for the attachment of their silken tube, such crevices and holes as they find on old walls, log cabins, &c., spreading from the door of their den long straggling threads to give the first warning of the approach of the heedless insect who stumbles against them. The females of these genera are most unrelentingly ferocious to the unfortunate individuals of the other sex; the trembling male approaches the abode of the objects of his desires "with cautious steps and slow"—trembling with fearful apprehension, whilst the female stands at her door, on the *qui vive*, ready to pounce upon and devour him at the first caprice of appetite or passion.

The following genera, *Lycosa* and *Dolomedes* are appropriately termed *Saltatores*, from the wild and active life they lead. They are the spirited *Nimrods* of the family of Araneides—wandering about without house or home, save at the time of incubation, 'their hand is against every man and every man's hand against them.' They leap with agility, and secure their prey by boldly chasing and subduing it. The female bears upon her back the cocoon filled with eggs, and when hatched, carries the young monsters for sometime, piled up in hideous profusion. She defends the cocoon with the utmost courage, taking it in her teeth, and never relinquishing her hold whilst life remains, though limb by limb be torn from her. If it be forcibly taken away she loses all animation and suffers herself to be destroyed without resistance.

The *Attus* is not less bold, but lacks the open fearlessness of the preceding. Like them it wanders about, never making a home except during incubation or hibernation, when it spins a small tent of finely woven silk, just large enough for one or two to enter. It seeks refuge in holes and chinks—travels boldly, running when alarmed, as well backwards as forwards. It steals upon its prey with stealthy, imperceptible advances, till within pouncing distance, when with a sudden spring and open jaws, it seizes and secures its victim, carrying it about in its mouth till the repast is completed.

The *Thomisus* is a clumsy looking, crab-like spider, crawling sideways, backwards or forwards with equal facility; the common place of abode for most species of this genus, is found in the cup of the sweetest flowers of Fall, where they lurk in ambush, feeding upon the insects attracted by the fragrance and sweets of the blossoms. During the bright, hazy days of Indian summer, this insect gathers in its long arm-like legs, a mass of flossy threads, and launches forth into the air, buoyed up by this light balloon and guided by a thread attached to its starting place, which it lets out like the string of a boy's kite, and with the spirit of a bold aeronaut, suffers the light breeze to waft it whithersoever it will.

The *Epeira* is the spider that spreads the beautiful polygonal or circular webs we see studded with dew drops on the bright mornings of spring, with a mathematical precision that would honor an Archimedes; often have I seen one, whilst toiling with the most indefatigable zeal, on finding that his circle was becoming an ellipse, retrace his steps for half the circumference and then return—putting in a *splice* as it were, to preserve the regularity of the web; they always, in stretching the circular threads, travel from the left to the right like the hands of a clock.

The *Mimetus* or *destroyer* as is indicated by the name, is the *highwayman* of the Araneides; roving about without any home of its own, plundering and pillaging every web that comes in its way, and after having devoured the inmate, taking quiet possession of its abode.

Thus we see that the apparently dull and insignificant spider is governed by instincts as curious and interesting as those we admire in insects of a more social and active character, but which are apt to escape our observation in this sedate and unobtrusive individual.

The gregarious bee or ant, whose astonishing sagacity has always excited the admiration and study of the observing mind, loses all its interest as a separate being—a single individual puts into play none of the intelligence which actuates him as a part of the community, and is exceedingly dull and helpless; whereas the solitary spider, fitted bv nature for leading an independent life, and relying for defence and subsistence on its own individual resources, displays, as remarked by Goldsmith, "actions which to me, who have attentively observed them, seem almost to exceed belief."

*The Spider's bite* is rarely productive of consequences serious enough to require the skill of the physician or surgeon—but as such a thing *may* happen, it is well to know what to expect and what to do upon such an occasion.

The symptoms which occur when the poison enters the wound, which does not appear always to be the case, and when the effects are severe, which is rarely or never the case save in feeble and cachectic individuals, are thus described:—"The sting is always accompanied with pain, and is followed by local phenomena, such as redness, œdematous or livid swelling, pustules, and general symptoms, such as numbness, cold, priapism, coma, convulsions and death itself." These effects are extreme and met with only in exceedingly rare cases; nevertheless that they do occur, we have evidence in many cases recorded by authority which we cannot doubt.

More astonishing effects are yet on record. Thus Martin Schurig reports a case of chlorosis following upon a bite; Comstock, a case of St. Vitus' dance; Berner and M. Serrieres, a malignant pustule which they believed to be produced by the bite of certain spiders.

Whenever the severity of the symptoms is such as to require the interposition of art, the following treatment will be found effective:—"Fomentation of the part, and refrigeration by means of iced water or evaporating lotions—emollient and narcotic applications—warm bathing of the part—oleaginous and opiated embrocations, and if the inflammation be very intense, local bloodletting must be employed. Compression by means of the bandage is an efficacious means of relief."

Should the violent symptoms of prostration above described, happen to ensue, they must be met by means of internal stimulation, the free administration of brandy, friction and heat to the extremities.

To illustrate what we may sometimes meet with in domestic practice, I will relate a case which happened at Saratoga in 1843. A boy 12 years of age was sent into the woods in the afternoon to drive in the cows. As he was strolling along he felt, as he thought, a brier prick his leg, and on looking down, saw a "large dark colored spider with big feet," on his pantaloons, which he brushed off and proceeded home, without paying much attention to it; however, he related the circumstance and complained of itching at night, three or four hours after the bite; a little red spot, without swelling, marked the spot; the pain rapidly increased as night advanced, physicians were called in, and saw no cause for the symptoms—no evidence of inflammation in the knee, which was the bitten part; they poulticed and cupped it, and gave opium internally. At three or four o'clock in the morning, they were called for again, and found the limb enormously tumefied, and the whole system affected with violent symptoms of prostration; all treatment proved unavailing, and convulsions and death closed the scene before morning. After death, the limb became speedily covered with purple blotches, the whole body became livid and discolored, and putrefaction advanced so rapidly that burial had to be performed on the same day.

We will conclude this department of our subject with a short description of the most venomous species which may be met with in our country.

There is a spider not often seen, but which may wound the foot of the plough-boy or gardener,—the *Mygale Truncata;* a huge and singularly shaped monster, about two or two and a half inches in length, the cephalo-thorax and legs of a deep piceous color, or nearly black, the first joint of the latter, rufous; a white membrane between the joints; the abdomen is singularly truncated, and marked with concentric grooves which give it a nutmeg appearance, and six circular impressions on its posterior or truncated face; the abdomen is callous at this extremity, and is supposed to act as a means of defence in plugging the mouth of its hole when attacked. It dwells in cylindrical holes, dug in the ground, to the depth of one and a half to two feet; and is frequently turned up in gardening, digging post-holes, &c. The bite is said to be exceedingly poisonous; a cat bitten by one, which was placed upon its back, died in a few minutes.

There is another subterranean species, whose bite is as severe, perhaps, as that of the preceding, the *Lycosa fatifera;* a large and stout species of a bluish black color; the legs are somewhat hairy, and there is at the base of the mandibulæ a red elevation, which adds to the ferocity of its appearance. It is quite a common spider, dwelling in holes of about a foot in depth, which are beautifully lined with silk, and surmounted at the edge of the opening with a rim, formed of bits of trash, nicely joined by interlacing threads, and covered with the same lining, but unfurnished with a lid which protects so accurately the dwellings of some species of the Mygale. If a straw be introduced into the hole and let down to the bottom, the manful resistance of the tenant can be immediately felt, and if carefully withdrawn, the spider may frequently be brought out, clinging to the straw with the most unyielding energy, holding it firmly in its teeth.

There is a large species of the *Mygale*, commonly known by the name *Tarantula*, which is found in the southern part of the Union, in Texas and Mexico; which must be exceedingly venomous, judging from the size of its fangs, which are fully as large as the claws of a grown cat. It grows to a very large size—with its legs spread, covering a surface of four or six inches; it is very hairy, and of a deep brown color. Of the nature of its bite, I know nothing.

Another spider, not so large as any of the preceding, but full of venom, is quite common in our country; the *Theridion verecundum*, shaped like and belonging to the same genus as the common house spider; its body attains the length of about three fourths of an inch; the whole insect is of a deep black color, with the exception of a bright red spot beneath the abdomen. Many varieties of this species have, along the longitudinal line of the back, two or three red spots, which are sometimes fringed with white.

It is found in the fields, in old stumps, hollow trees, fence corners, &c., hiding in some crevice, whence it spreads a scattering web of remarkable strength, which enables it to entrap the largest insects. I have no experience with regard to its bite, but it is said to be quite venomous, and judging from its vigor, the strength of its web, and the size of the insects which it captures, I think the conclusion a probable one.

The last portion of the subject, and that which has most to do with its acceptance as a medical Thesis, is the employment of the web of the spider, as an article of the materia medica, under the name of *Tela Aranearum.*

Other products of the spider, besides its web, have been used as medicinal agents.

The insect itself has been employed, internally and externally, from remote antiquity. Externally it has been applied to the wrists, mashed into a kind of poultice, to prevent the access of fevers. Etmuller has employed it, dried and pulverized, in doses of gr. xx or xxx, as an anti-periodic. As an aphrodisiac, it has been celebrated in olden time; Lorry relates a striking example of its effects in this way, and the people of Kamtschatka place great faith in its fecundating qualities. In Chili, according to Molino, there is a fat spider, as large as a hen's egg, which the children use as a plaything, and which is employed as a remedy for the tooth-ache.

The eggs have been recommended also for the cure of tooth-ache by Archigenus; the water distilled from black spiders, by Lister, in the treatment of wounds; oils have been prepared from them, for the cure of ulcers and malignant inflammation; an empyreumatic oil has been extracted from them, for the cure of warts, &c.

With nothing more than a mere enumeration of these chimerical relics of the dark ages of medicine, we will turn our attention, in conclusion, to the use of its web alone.

Externally, it is a popular and efficient application as an hæmostatic—acting I presume, not from any specific virtue in this way, but from the softness of its texture, which becomes soon glued down by the coagulation of the blood in its meshes. It is also employed by some surgeons for covering arsenical paste and other escharotic applications to the surface.

But it is chiefly entitled to our consideration as an internal medicine; its efficacy as an antiperiodic, and quietus of the nervous system, has been testified to by men of the highest professional standing, and deserves, in my humble opinion, more attention from the members of our profession than it has received.

Dr. Robert Jackson, in the Medical and Physical Journal of May 1809 (Vol. XXI, No. 123) gives in detail, the results of his experiments with it; he was led to its trial, from being told by Dr. Gillespie, of Edinburgh, that he had cured by its use, an obstinate intermittent that had withstood every other treatment.

He relates several cases of intermittent fever, in which he used it with the most perfect success: First, in the person of a respectable soldier, who had long been troubled by the disease, which though stopped by the use of bark and arsenic, was ever recurring. He administered a five grain pill, two hours before the expected chill, and again, two more of them only a few moments before the time for the paroxysm. In this, and three other cases similarly treated, the usual periods passed over, without the slightest indication of the chill, and the disease remained cured; the influence of the imagination could have had no effect in any of the cases recorded, for none of the patients were aware of the nature of the remedy employed.

Encouraged by this success, he gave it in another case, in the midst of the paroxysm, whilst the patient was in a state of deadly collapse, from which, he most unexpectedly recovered, "in less than a minute," as if by a charm; the pulse becoming "calm and orderly, a very gentle, but warm perspiration taking place immediately, and health being instantly restored."

From these decisive experiments, he came to the conclusion "that cobweb possesses an extraordinary, and altogether inexplicable power in calming irritations, and in diminishing excess of bodily torments," and was further induced to try it in other forms of febrile irritation—viz: "in the deliria, pains, spasms, and subsultus tendinum in fevers of the continued class, and having done so in various instances, was warranted in saying that the effect far exceeded his expectations; it gave tranquillity and sometimes, perhaps saved life."

He continued his trials, and "effected perfect cures in some troublesome spasmodic affections, and found that respite was procured in others that were fatal in themselves, and that seemed to stand beyond the reach of other means." He cites in point, a case of phthisis, and one of complicated organic disease, in which it proved a most excellent palliative, tranquillizing irritation, and producing a happy state of narcotism, not followed by any of the disagreeable results that follow on the administration of opium.

In conclusion he suggests that it be tried in hydrophobia, that dreadful and as yet incurable malady, which is "a disease of excessive irritability, and cobweb diminishes morbid irritability, and calms irritations of both body and mind, in a degree far exceeding any drug or medicine within the circle of our knowledge."

The web which is to be employed, is that of the *Tegenaria medicinalis, or Agalena naevia*, a wide hammock-like web, found in the corners of cellars, old dry-goods boxes, and other dark places. A description of this spider, by my father, together with a drawing and allusion to its medicinal use, is to be found in the Journal of the Academy of Natural Sciences of Philadelphia, (Vol. 11, p. 53, plate v. fig. 1.)

